# HapMap-based study of the 17q21 *ERBB2* amplicon in susceptibility to breast cancer

**DOI:** 10.1038/sj.bjc.6603473

**Published:** 2006-11-21

**Authors:** P R Benusiglio, P D Pharoah, P L Smith, F Lesueur, D Conroy, R N Luben, G Dew, C Jordan, A Dunning, D F Easton, B A J Ponder

**Affiliations:** 1 Strangeways Research Laboratory, Cancer Research UK Department of Oncology, University of Cambridge, Strangeways Research Laboratory, Worts Causeway, Cambridge CB1 8RN, UK; 2 Department of Internal Medecine, Hôpital Cantonal Universitaire de Genève, 24 rue Micheli-du-Crest, 1211 Genève 14, Switzerland; 3 Department of Genetic Epidemiology, University of Cambridge, Strangeways Research Laboratory, Worts Causeway, Cambridge CB1 8RN, UK; 4 Génomes et Cancers, Institut de Cancérologie Gustave Roussy, rue Camille Desmoulins, 94805 Villejuif, France; 5 EPIC, University of Cambridge, Strangeways Research Laboratory, Worts Causeway, Cambridge CB1 8RN, UK

**Keywords:** breast neoplasms, *ERBB2*, amplicon, genetic susceptibility, single-nucleotide polymorphism, HapMap

## Abstract

*ERBB2* is frequently amplified in breast tumours as part of a wide region of amplification on chromosome 17q21. This amplicon contains many candidate genes for breast cancer susceptibility. We used a genetic association study design to determine if common genetic variation (frequency ⩾5%) in a 400-kb region surrounding *ERBB2* and containing the *PPARBP*, *CRK7*, *NEUROD2*, *PPP1R1B*, *STARD3*, *TCAP*, *PNMT*, *CAB2*, *ERBB2*, *C17ORF37*, *GRB7* and *ZNFN1A3* genes, was associated with breast cancer risk. Sixteen tagging single-nucleotide polymorphisms (tSNPs) selected within blocks of linkage disequilibrium from the HapMap database, one HapMap singleton SNP, and six additional SNPs randomly selected from dbSNP were genotyped using Taqman in a large study set of British women (2275 cases, 2280 controls). We observed no association between any of the genotypes or associated haplotypes and disease risk. In order to simulate unidentified SNPs, we performed the leave-one-out cross-validation procedure on the HapMap data; over 90% of the common genetic variation was well represented by tagging polymorphisms. We are therefore likely to have tagged any common variants present in our population. In summary, we found no association between common genetic variation in the 17q21 *ERBB2* amplicon and breast cancer risk in British women.

Only a small proportion of the excess familial risk associated with breast cancer is accounted for by known highly penetrant genes, *BRCA1* and *BRCA2.* The remainder is probably due to a combination of weakly predisposing alleles including both common and rare variants (Pharoah *et al*, 2002; Dite *et al*, 2003). We have previously shown that common alleles in *ERBB2* are not involved in breast cancer susceptibility (Benusiglio *et al*, 2005). However, *ERBB2* is part of a wider region of chromosome 17q21 frequently amplified in breast cancer. This amplicon encompasses many genes and it is conceivable that, as suggested by the variability of response to anti-*ERBB2* therapy in patients with *ERBB2* amplification, more than one gene in the amplicon could contribute to breast cancer susceptibility, development and progression (Willis *et al*, 2003).

Kauraniemi *et al* (2001, 2003) first carried out a systematic survey of copy number and expression patterns in all genes within the 17q21 locus on breast cancer cell lines and tumour samples; they identified a 200-kilobase (kb) minimal common region of amplification around *ERBB2* containing the following genes: neurogenic differentiation 2 (*NEUROD2*), protein phosphatase 1 regulatory subunit 1B (*PPP1R1B*), START domain containing 3 (*STARD3*), titin-cap (*TCAP*), phenylethanolamine-*N*-methyltransferase (*PNMT*), *CAB2* (Per1-Like Domain Containing 1 [*PERLD1*]), *C17ORF37*, growth factor receptor-bound protein 7 (*GRB7*) and zinc-finger protein subfamily 1A 3 (*ZNFN1A3*).

Gene expression levels in cancer cells together with knowledge about protein function can help in assessing which genes are involved in oncogenesis (Futreal *et al*, 2004). For example, *STARD3*, *PNMT*, *CAB2*, *C17ORF37* and *GRB7* show a significant correlation between amplification status and expression level (Kauraniemi *et al*, 2001, 2003; Willis *et al*, 2003; Orsetti *et al*, 2004), and *STARD3*, *PNMT*, *CAB2*, *GRB7* and *ZNFN1A3* could all be biologically relevant to breast cancer. *STARD3* mediates intracellular trafficking of cholesterol and can augment steroid hormone synthesis (Strauss, III *et al*, 2003). Overexpression of *PNMT* results in suppression of circulating leptin levels – a potent regulator of body weight – in transgenic mice (Bottner *et al*, 2000; Harvey and Ashford, 2003). *CAB2* is a human homologue of the yeast *COS16* gene required for the repair of DNA double-strand breaks (Nezu *et al*, 2002) and *GRB7* regulates cell migration through its involvement in cell signalling pathways (Han *et al*, 2001). Finally, *ZNFN1A3* appears to function as a tumour suppressor since its downregulation in the mouse leads to leukaemias and lymphomas (Rebollo and Schmitt, 2003).

Two neighbouring genes, peroxisome proliferator-activated receptor binding protein (*PPARBP*) and CDC2-related protein kinase 7 (*CRK7*), located about 50 kb upstream from *NEUROD2* – the first gene on the minimum region of amplification – are often, although less consistently, co-amplified with *ERBB2* (Kauraniemi *et al*, 2001, 2003). Both are potentially implicated in cancer biology. *PPARBP*, by its ability to function as an oestrogen receptor coactivator, might play a role in mammary epithelial differentiation (Zhu *et al*, 1999) while *CRK7* could link transcription with the splicing machinery (Ko *et al*, 2001).

The case–control study design is well suited to the identification of small-effect genes that are likely to underlie common, complex diseases such as breast cancer (Risch, 2000). Two approaches have been proposed. The direct, hypothesis-driven approach is to investigate single nucleotide polymorphisms (SNPs), which are thought to have functional effects and thus influence directly the traits under study (Tabor *et al*, 2002). The indirect, tagging approach is to select a set of empirical tagging SNPs (tSNPs) that best capture the common genetic variation within the gene. They serve as markers to detect associations between a particular region and diseases, whether or not the tSNPs themselves have a functional effect (Gabriel *et al*, 2002). It is not necessary to genotype all polymorphisms because the alleles of SNPs that are physically close to each other tend to be correlated with each other: they are in linkage disequilibrium (LD) (Pharoah *et al*, 2004). The HapMap online database (http://www.hapmap.org) allows the tagging approach to be applied readily to many genes or regions (Gibbs *et al*, 2003). By March 2006, the database held the genotypes of individuals with European, African-American, and Asian ancestry for nearly four million SNPs.

We aimed to determine whether common genetic variation (frequency >5%) in the *ERBB2* amplicon is involved in breast cancer susceptibility. We used HapMap data to identify tSNPs for genotyping in a large breast cancer case–control study of white British women. Data for five of the SNPs described in this report have been previously published (Benusiglio *et al*, 2005), but are also included here for completeness.

## METHODS

### Patients and controls

Cases were drawn from SEARCH, an ongoing population-based study in which cases are ascertained through the East Anglian Cancer Registry. All patients diagnosed with invasive breast cancer below age 55 years since 1991 and still alive in 1996 (prevalent cases; median age 48 years), together with all those diagnosed <70 years between 1996 and the present (incident cases; median age 52 years), were eligible to take part. All study participants completed an epidemiological questionnaire and provided a blood sample for DNA analysis. Sixty-seven percent of eligible breast cancer patients returned a questionnaire and 64% provided a blood sample. Controls were randomly selected from the Norfolk component of EPIC (European Prospective Investigation of Cancer). EPIC is a prospective study of diet and cancer being carried out in nine European countries. The EPIC-Norfolk cohort comprises 25 000 individuals resident in Norfolk, East Anglia, the same region from which the cases have been recruited. Controls are not individually matched to cases, but are broadly similar in age, being aged 42–81 years. The ethnic background of both cases and controls as reported on the questionnaires is similar, with >98% being white. All participants have given written consent and the study is approved by the Eastern Region Multicentre Research Ethics Committee.

A total of 4474 cases, of whom 27% were prevalent cases, and 4560 controls, were available for analysis. The samples have been split into two sets in order to conserve DNA and reduce genotyping costs. The first set (2275 cases, 2280 controls) is genotyped for all SNPs. Any SNP that shows association in set 1 at the *P*<0.1 level, can then be tested in the second set (2199 cases, 2280 controls). This staged approach substantially reduces genotyping costs without significantly affecting statistical power (see below). Cases with high yields of genomic DNA were selected for set 1 from the first 3500 recruited, with set 2 comprising the remainder of these plus the next 974 incident cases recruited. As the prevalent cases were the first recruited, the proportion of prevalent cases was somewhat higher in set 1 than set 2 (33 *vs* 20%). Median age at diagnosis was similar in both sets (51- and 52-year-old, respectively). Median time from diagnosis to blood draw was slightly longer for set 2 (15 months) than for set 1 (9 months). There were no significant differences in the morphology, histopathological grade or clinical stage of the cases by set or by prevalent/incident status.

### SNP identification and selection

**Figure 1:**
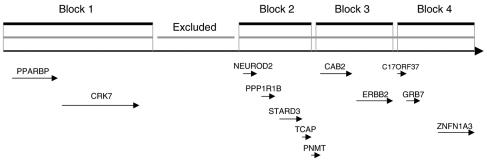
The 400-kb *ERBB2* amplicon. It is split in two (regions **A** and **B**) by a 50-kb segment containing no known gene. Region **A** consists of one LD block while region **B** consists of three blocks, blocks 2, 3 and 4.

The amplicon of interest is a 400-kb region, with *PPARBP* in position 5′ and *ZNFN1A3* in position 3′ (Figure 1). A 50-kb segment located between *CRK7* and *NEUROD2* was excluded as it contained no known gene, splitting the amplicon into a 150-kb region including *PPARBP* and *CRK7* (region A) and a 200-kb region including *NEUROD2*, *PPP1R1B*, *STARD3*, *TCAP*, *PNMT*, *CAB2*, *ERBB2*, *C17ORF37*, *GRB7* and *ZNFN1A3* (region B).

We used data on common SNPs from HapMap (European samples, public release #15) to identify tSNPs. After exclusion of one singleton SNP that was poorly linked with any other SNP (D′ <0.3), blocks of LD were defined on the basis of limited haplotype diversity (common haplotypes must account for at least 90% of all haplotypes) (Cardon and Abecasis, 2003). tSNPs were selected within blocks using the tagSNPs program so that unmeasured SNPs were tagged with a minimum *r*_s_^2^ of 0.8 (Stram, 2004). The *r*_s_^2^ coefficient measures LD between unmeasured SNPs and haplotypes defined by the selected tSNPs. The five *ERBB2* SNPs previously genotyped (ERBB2-01 to -05) were forced as tSNPs into the selection algorithm and SNPs surrounded by repetitive DNA sequences were excluded (they could not be selected as tSNPs). The average SNP density of the HapMap SNPs was 1 SNP/5 kb. In order to increase the SNP density and improve the tagging properties of our SNP set, we also selected at the beginning of the study additional random, validated polymorphisms from the dbSNP database (http://www.ncbi.nlm.nih.gov/projects/SNP).

### Genotyping

Genotyping was carried out using Taqman (Applied Biosystem, Warrington, UK). Primers and probes were supplied directly by Applied Biosystems, except those for ERBB2-04 and -05 that were designed ‘in-house’ with the Primer Express Oligo Design Software v2.0 (Applied Biosystems). Sequences are available on request. Reactions were carried out at 54 or 60°C in 384-well plates with cases and controls plated together. Each plate included two negative controls with no DNA and 12 samples duplicated on a separate quality control plate. Plates were read on the ABI Prism 7900 using the Sequence Detection Software (Applied Biosystems). Complete concordance between samples and their duplicates, excluding undetermined genotypes, was required for the assay to be validated. Failed genotypes were not repeated.

### Statistical methods

For each SNP, deviation of genotype frequencies in controls from Hardy–Weinberg (HW) equilibrium was assessed by a standard *χ*^2^ test (1 degree of freedom (df)). Genotype frequencies in cases and controls were compared by a *χ*^2^ test for heterogeneity (2 df). Genotype-specific risks were estimated as odds ratios (OR) using standard cross-product ratio and confidence intervals were calculated using the variance of the log (OR), which was estimated by the standard Taylor expansion. A comparison of haplotype frequencies between cases and controls was carried out using the haplo.score routine implemented in S-plus (Schaid *et al*, 2002). Haplotypes with an estimated frequency of <5% were pooled. Haplo.score uses a likelihood that depends on estimated haplotype frequencies to test the statistical association between haplotypes and phenotype. It is based on score statistics, which provide both global tests and haplotype-specific tests.

## RESULTS

The HapMap release #15 included genotypes for 66 common SNPs (26 in region A and 40 in region B). Region A consisted of only one LD block, block 1 with 26 SNPs, whereas region B consisted of three blocks, block 2 with 14 SNPs, block 3 with 11 SNPs and block 4 with 15 SNPs. One singleton SNP, ERBB2-04 (I655V), was excluded from block 3, leaving 10 SNPs in block 3. Block 1 included *PPARBP* and *CRK7*, block 2 included *NEUROD2*, *PPP1R1B*, *STARD3* and *TCAP*, block 3 included *CAB2* and *ERBB2* and block 4 included *C17ORF37*, *GRB7* and *ZNFN1A3* (Figure 1).

**Figure 2:**
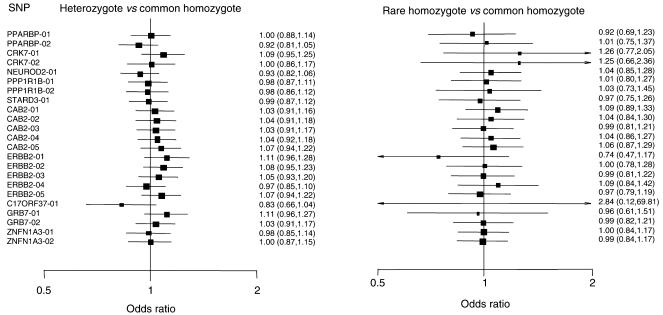
Genotype-specific risks for 23 SNPs genotyped in 2275 women with breast cancer and 2280 controls.

Sixteen tSNPs were selected for genotyping: four in block 1, three in block 2, five in block 3 and four in block 4 (Table 1). ERBB2-04 - the HapMap singleton SNP – and six additional SNPs randomly selected from dbSNP (one in block 2, four in block 3 and one in block 4) were also genotyped. None of the genotype distributions in controls differed significantly from those expected under HW equilibrium. There was no evidence that any of the SNPs is associated with breast cancer, and none of the SNPs exceeded the significance threshold for genotyping in the second set of cases and controls (Table 2). The genotype-specific ORs were all close to unity with confidence intervals including one (Figure 2). The tSNPs generated five common haplotypes in block 1, three in block 2, four in block 3 and four in block 4 (Table 3). The global test of association was not significant for any of the four blocks (*P*=0.16, 0.58, 0.48 and 0.44 for blocks 1, 2, 3 and 4, respectively), nor were there any differences between cases and controls for the individual haplotype frequencies.

## DISCUSSION

We used a comprehensive SNP tagging approach using the publicly available HapMap data, complemented by the random selection of SNPs from dbSNP, to determine if common variation in the *ERBB2* amplicon is involved in breast cancer susceptibility. A total of 23 SNPs in 4 LD blocks were genotyped; we saw no association between any of the genotypes or associated haplotypes and risk of breast cancer.

We could have failed to observe a true association because of inadequate tagging, insufficient statistical power, or the effect of confounders. The selection of tagging SNPs is most reliable where the region of interest has been resequenced in a sample of individuals sufficiently large to identify all common variants. Such data were not available for this region, and it is possible that we have not adequately tagged an unidentified, disease-predisposing SNP. We estimated how well tSNPs would tag such unknown SNPs by performing a leave-one-out cross validation procedure on the HapMap data used for tSNP selection. Each of the 65 known SNPs were dropped in turn and tSNPs selected from the remaining SNPs within the block, thus simulating unidentified polymorphisms (Ahmadi *et al*, 2005). The ability of the tSNPs to tag the dropped SNP was then evaluated by calculating *r*_s_^2^. The average *r*_s_^2^ for all dropped SNPs was 0.91 and 59 out of the 65 dropped SNPs (91%) were tagged with an *r*_s_^2^>0.75. This suggests that 91% of the unknown variation was well tagged. Furthermore, it has been shown that Phase 2 HapMap data provides a robust alternative to complete re-sequencing data with minimal loss of power (De Bakker *et al*, 2005). After we had completed the genotyping for this study, data for phase 2 of the HapMap project were released. For region A, there were now data for 35 SNPs – SNP density 1 SNP/4.2 kb. We used the programme TAGGER to test the performance of our selected tSNPs on HapMap phase 2 (Paul de Bakker, http://www.broad.mit.edu/mpg/tagger/). Mean pairwise *r*^2^ (*r*^2^_p_) was 0.92 with 31 HapMap SNPs being tagged with *r*^2^_p_>0.8 and a minimum *r*^2^_p_ of 0.21. For region B there were data on 57 SNPs (1 SNP/3.3 kb). Mean *r*^2^_p_ was 0.91 with 53 HapMap SNPs being tagged with *r*^2^_p_>0.8 and a minimum *r*^2^_p_ of 0.12. These are conservative estimates since additional SNPs with undetermined tagging properties were randomly selected from dbSNP. Thus, the majority of common genetic variation is likely to have been captured, but we cannot exclude the possibility that an important common variant was missed.

*PNMT* is thought to regulate leptin levels and could be relevant to breast cancer biology via its effect on body weight (Harvey and Ashford, 2003; Stattin *et al*, 2004). None of the HapMap SNPs were in *PNMT*, raising questions regarding appropriate tagging of the gene. Ahmadi *et al* (2005) studied tagging patterns across 55 genes, including *PNMT*, that control the absorption, distribution, metabolism and excretion of drugs; they were able to determine that variation within *PNMT* was adequately tagged by rs1053651 in *TCAP* and rs903502 in *CAB2*, two HapMap SNPs that were well tagged by our set of tSNPs.

The statistical power of the study depends on the risk allele frequency, the risks conferred and the genetic mode of action (dominant, recessive, codominant). The staged approach substantially reduces genotyping costs without significantly affecting statistical power. For example, assuming that the causative SNP is tagged with *r*^2^_p_=0.8, a type I error rate of 0.0001 and genotyping success rate of 0.95, the staged study has 86% power to detect a dominant allele with a minor allele frequency (MAF) of 0.05 that confers a relative risk of 1.5 or 87% power to detect a dominant allele with a MAF of 0.25 that confers a relative risk of 1.3. Power to detect recessive alleles is less – 53% for an allele with a MAF of 0.25 and a relative risk of 1.5 and 71% for an allele with a MAF of 0.5 and a relative risk of 1.3. Such high power is illustrated by the narrow confidence intervals observed for odds ratios associated with genotypes: heterozygote odds ratios higher than 1.28 were excluded for all SNPs. Based on the upper confidence limits for all the risk estimates we can exclude SNPs that explain more than 0.8% of the excess familial risk of breast cancer. The possibility of variants with smaller effects on risk cannot be excluded. Similarly the possibility that there are rare variants with modest, or even large effect cannot be excluded.

Confounders are factors that are associated with both genotype and phenotype. They may bias results towards false positives and false negatives. In the context of breast cancer genetic association studies, it is difficult to envisage a true confounder. Most factors that are likely to be associated with both genotype and breast cancer will be intermediate factors, not confounders. The cases and controls used for these analyses were not matched for age (though broadly similar). However, there was no association of genotype with age in controls for any of the SNPs studied and age-adjusted odds ratios were similar to the unadjusted values.

Some authors have advocated the use of histopathologic or demographic data that subclassify individuals in order to identify homogeneous subsets for analysis (Rebbeck *et al*, 2004). In the absence of any main effect or strong biological rationale, we have not carried out subgroup analyses. The number of possible *post hoc*, subgroup analyses is large and there is a strong possibility that one or more tests will be statistically significant simply by chance (Colhoun *et al*, 2003; Pharoah *et al*, 2005). Much larger sample sizes would be required to obtain reliable results. Nor did we test for SNP-SNP or SNP-environment interactions as the number of such interactions is very large – there are over 2000 possible two- and three-way interactions between the 23 genotyped common SNPs – and a clear strategy on how to best approach interactions has yet to be defined (Ritchie *et al*, 2003; Hunter, 2005).

We found no evidence that common genetic variation in the *ERBB2* amplicon is associated with an altered risk of breast cancer. The strategy of selecting candidate genes from regions that are often amplified was not successful here, but we have only evaluated a small proportion of the genome that is commonly somatically altered in breast cancer. The search for common susceptibility alleles for breast and the other common cancers using a candidate gene approach has been notable for its lack of success. However, there are many, possibly thousands, of candidate genes of which only tens or hundreds have been comprehensively assessed for susceptibility. Advances in our understanding of human genomic architecture with rapid developments in high-throughput genotyping technology have made empirical, genome-wide association (GWA) studies feasible. The results of the first GWA scans in breast and colorectal cancer are expected in the near future. These should provide evidence whether or not common susceptibility variants exist. They may also provide an indication whether candidate gene studies remain a valid approach and, if so, what the likely candidates are.

## Figures and Tables

**Table 1:** SNPs selected for genotyping, the database they were selected from, the LD block to which they belong, their location within genes and their frequencies in databases

**SNP**	**rs number**	**Database**	**Block**	**Location**	**Base change**	**MAF**
PPARBP-01	rs6503513	HapMap	1	START −1240	a>g	0.16
PPARBP-02	rs11655550	HapMap		STOP +2808	t>c	0.24
CRK7-01	rs2303315	HapMap		IVS10 +111	t>a	0.13
CRK7-02	rs4404103	HapMap		IVS13 +88	a>g	0.12
						
NEUROD2-01	rs12453682	HapMap	2	STOP +5832	t>c	0.28
PPP1R1B-01	rs1874228	HapMap		START −8372	g>a	0.24
PPP1R1B-02	rs879606	HapMap		START −1797	g>a	0.13
STARD3-01	rs3817160	dbSNP		IVS1 +331	c>g	0.45
						
CAB2-01	rs2952151	HapMap	3	START −560	c>t	0.28
CAB2-02	rs907087	dbSNP		START −269	a>g	0.46
CAB2-03	rs1565920	dbSNP		IVS5 +681	a>g	0.38
CAB2-04	rs907089	dbSNP		IVS5 +2668	a>g	0.39
CAB2-05	rs1476278	HapMap		IVS5 +5311	a>g	0.29
ERBB2-01	rs4252596	dbSNP		START −657	c>a	0.07
ERBB2-02	rs2952155	HapMap		IVS1 +5154	c>t	0.2
ERBB2-03	rs1810132	HapMap		IVS4 +300	t>c	0.28
*ERBB2-04*	*rs1801200*	*HapMap*		*EX17 (I655 V)*	*a*>*g*	*0.33*
ERBB2-05	rs1058808	HapMap		EX27 (A1170P)	g>c	0.29
						
C17ORF37-01	rs4252665	HapMap	4	START −26	c>t	0.06
GRB7-01	rs8192704	HapMap		IVS2 +13	g>a	0.16
GRB7-02	rs11078921	dbSNP		STOP +5330	c>a	0.26
ZNFN1A3-01	rs907091	HapMap		START −361	c>t	0.47
ZNFN1A3-02	rs10445308	HapMap		IVS2 +4027	c>t	0.48

Sixteen SNPs were HapMap tSNPs, one was a HapMap singleton (ERBB2-04) and six were randomly selected from the dbSNP database.

**Table 2:** Genotype frequencies, minor allele frequencies (MAF) and *P*-values for 23 SNPs genotyped in 2275 women with breast cancer and 2280 controls

**SNP**	**Series**	**Number genotyped**	**MAF**	**Common homozygote**	**Heterozygote**	**Rare homozygote**	***P*-value^a^**
PPARBP-01	Cases	2190	0.20	1422	665	103	0.85
	Controls	2274		1481	694	99	
PPARBP-02	Cases	2181	0.19	1421	675	85	0.47
	Controls	2273		1516	665	92	
CRK7-01	Cases	2186	0.14	1658	499	29	0.36
	Controls	2273		1685	551	37	
CRK7-02	Cases	2193	0.10	1797	379	17	0.79
	Controls	2275		1859	394	22	
NEUROD2-01	Cases	2180	0.31	1020	941	219	0.4
	Controls	2271		1090	937	244	
PPP1R1B-01	Cases	2150	0.27	1152	833	165	0.95
	Controls	2245		1210	860	175	
PPP1R1B-02	Cases	2085	0.17	1435	585	65	0.94
	Controls	2198		1520	607	71	
STARD3-01	Cases	2158	0.22	1303	736	119	0.96
	Controls	2266		1377	767	122	
CAB2-01	Cases	2184	0.32	1042	923	219	0.71
	Controls	2276		1064	969	243	
CAB2-02	Cases	1923	0.31	930	801	192	0.84
	Controls	2027		961	859	207	
CAB2-03	Cases	2179	0.33	995	953	231	0.85
	Controls	2274		1024	1014	236	
CAB2-04	Cases	2185	0.34	961	969	255	0.78
	Controls	2269		974	1025	270	
CAB2-05	Cases	2038	0.35	901	890	247	0.58
	Controls	2157		919	971	267	
ERBB2-01	Cases	2023	0.13	1548	433	42	0.14
	Controls	2189		1645	511	33	
ERBB2-02	Cases	2040	0.26	1162	738	140	0.45
	Controls	2205		1219	839	147	
ERBB2-03	Cases	2050	0.32	969	861	220	0.69
	Controls	2208		1022	956	230	
ERBB2-04	Cases	1999	0.25	1134	752	113	0.67
	Controls	2154		1229	791	134	
ERBB2-05	Cases	2025	0.33	916	875	234	0.47
	Controls	2180		960	982	238	
C17ORF37-01	Cases	2169	0.03	1997	172	0	0.16
	Controls	2259		2108	150	1	
GRB7-01	Cases	2188	0.13	1676	474	38	0.36
	Controls	2273		1703	533	37	
GRB7-02	Cases	2171	0.34	954	969	248	0.85
	Controls	2269		983	1032	254	
ZNFN1A3-01	Cases	2088	0.49	561	1002	525	0.97
	Controls	2214		600	1055	559	
ZNFN1A3-02	Cases	2172	0.48	589	1086	497	0.99
	Controls	2278		620	1140	518	

a Test for heterogeneity of genotype frequencies between cases and controls (2 df).

**Table 3:** Haplotype frequencies within LD blocks. ERBB2-04, a HapMap singleton SNP, was excluded from block 3 for haplotype analyses as it is poorly correlated with any other SNPs in the block

**Block**	**Global test of association**	**Haplotype**	**Frequency in controls (%)**	***P*-value**
1	0.16	-a-t-t-a-	41	0.74
		-a-c-t-a-	19	0.43
		-g-t-t-a-	16	0.24
		-a-t-a-a-	13	0.13
		-a-t-t-g-	7	0.48
				
2	0.58	-t-g-g-c-	68	0.8
		-c-a-a-g-	17	0.96
		-c-a-g-c-	7	0.41
				
3	0.48	-c-a-a-a-a-c-c-t-g-	51	0.34
		-t-g-g-g-g-c-t-c-c-	24	0.24
		-c-a-a-a-a-a-c-t-g-	13	0.31
		-t-g-g-g-g-c-c-c-c-	6	0.57
				
4	0.44	-c-g-c-t-c-	37	0.55
		-c-g-a-c-t-	28	0.75
		-c-g-c-c-t-	13	0.27
		-c-a-c-t-c-	9	0.19
